# Impaired pain sensation in mice lacking prokineticin 2

**DOI:** 10.1186/1744-8069-2-35

**Published:** 2006-11-15

**Authors:** Wang-Ping Hu, Chengkang Zhang, Jia-Da Li, Z David Luo, Silvia Amadesi, Nigel Bunnett, Qun-Yong Zhou

**Affiliations:** 1 Department of Pharmacology, University of California, Irvine, CA 92697, USA; 2 Department of Anesthesiology, University of California, Irvine, CA 92697, USA; 3 Departments of Surgery and Physiology, University of California, San Francisco, CA 94143, USA

## Abstract

Prokineticins (PKs), consisting of PK1 and PK2, are a pair of newly identified regulatory peptides. Two closely related G-protein coupled receptors, PKR1 and PKR2, mediate the signaling of PKs. PKs/PKRs participate in the regulation of diverse biological processes, ranging from development to adult physiology. A number of studies have indicated the involvement of PKs/PKRs in nociception. Here we show that PK2 is a sensitizer for nociception. Intraplantar injection of recombinant PK2 resulted in a strong and localized hyperalgesia with reduced thresholds to nociceptive stimuli. PK2 mobilizes calcium in dissociated dorsal root ganglion (DRG) neurons. Mice lacking the *PK2 *gene displayed strong reduction in nociception induced by thermal and chemical stimuli, including capsaicin. However, *PK2 *mutant mice showed no difference in inflammatory response to capsaicin. As the majority of PK2-responsive DRG neurons also expressed transient receptor potential vanilloid (TRPV1) and exhibited sensitivity to capsaicin, TRPV1 is likely a significant downstream molecule of PK2 signaling. Taken together, these results reveal that PK2 sensitize nociception without affecting inflammation.

## Background

Prokineticins (PKs), consisting of PK1 and PK2, are a novel family of regulatory peptides, whose mature forms consist of 86 and 81 amino acids, respectively [1]. Both PK1 and PK2 possess ten conserved cysteines and have about 45% identity in the amino acid sequences [2]. Two endogenous G-protein coupled receptors for PKs, PKR1 and PKR2, have been identified in humans, rats and mice [3-5]. PKR1 and PKR2 are highly similar to each other and appear to signal mainly through Gq pathway [3].

Several regulatory functions ranging from development to adult physiology have been described for PKs [1,6-9]. A number of studies have indicated the involvement of the PKs/PKRs system in nociception. Intraplantar injection of Bv8, the frog homolog of PKs, causes a strong and localized hyperalgesia by reducing the nociceptive thresholds to thermal and mechanical stimuli [10,11]. The hyperalgesia caused by Bv8 is likely due to the sensitization of transient receptor potential vanilloid 1 (TRPV1) in dorsal root ganglion (DRG) neurons [12]. Although both PKR1 and PKR2 are expressed in the DRG neurons, a recent report that mice lacking the *PKR1 *gene exhibit impaired pain perception to various stimuli including noxious heat, mechanical, capsaicin, and protons implies that PKR1 is likely to be the dominant receptor that exerts a tonic activation of TRPV1 [13].

Whether PK1 or PK2 is responsible for the activation of PKR1 in pain sensitization is unknown. PK2 is highly expressed in peripheral blood cells, notably in monocytes, neutrophils, and dendritic cells. PK2 has been identified as a chemoattractant for monocyte/macrophage [9,14]. At the sites of inflammation, neutrophils may release PK2 that can subsequently induce the release of proinflammatory cytokines like interleukin-1 and interleukin-12 from macrophage or other cells [15]. In contrast, PK1 was not found in the peripheral blood cells [9]. Thus, even though PK1 activates PKR1 with similar potency in vitro as PK2 [3], PK2 might be the dominant ligand involved in nociception, especially in inflammatory pain. In the present study, we explored the involvement of PK2 in pain sensation. Our studies reveal that PK2 is a sensitizer for inflammatory pain without affecting inflammation.

## Results

### Nociceptive sensitization to thermal stimuli by intraplantar injection of PK2

**Figure 1:**
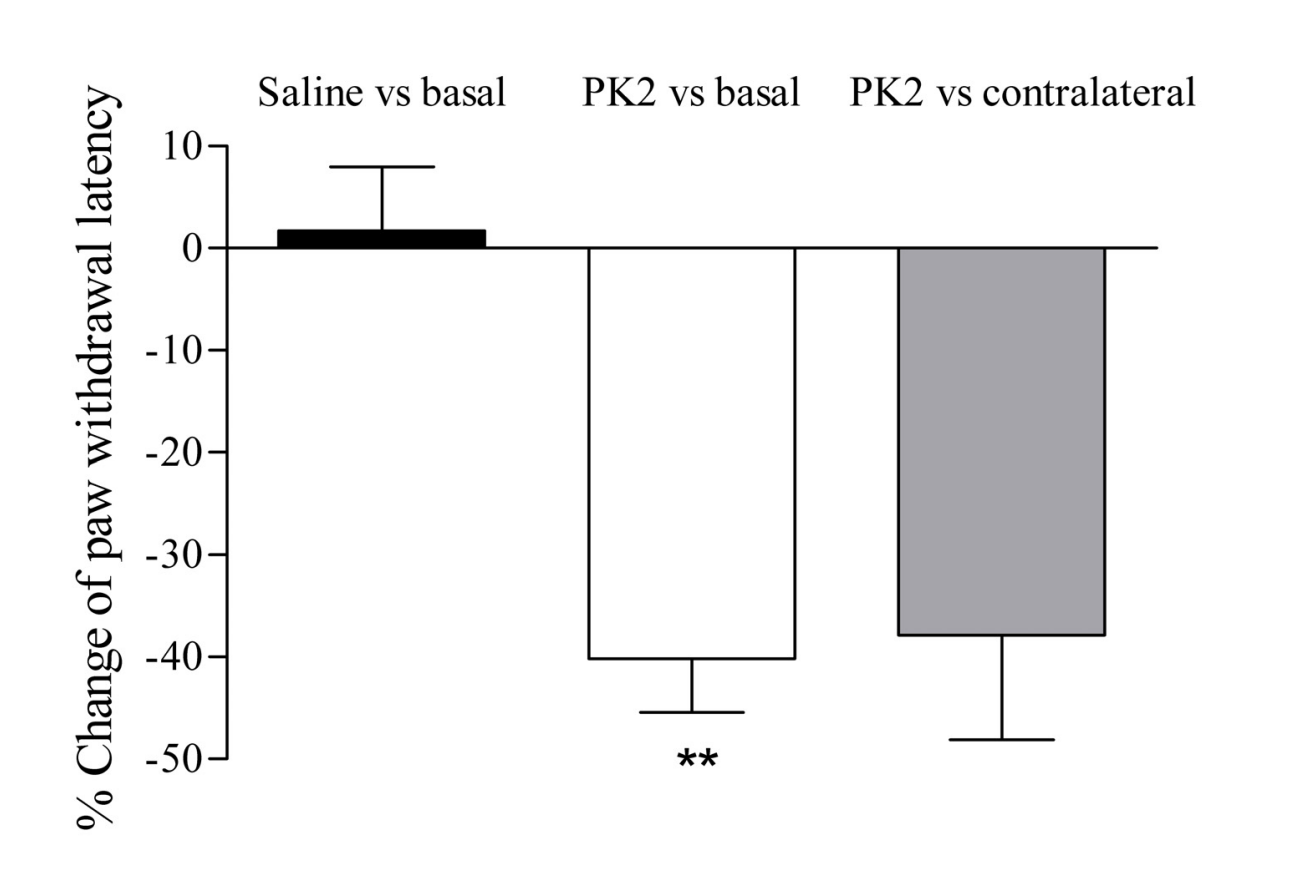
Heat hyperalgesia induced by intraplantar injection of PK2. Paw withdrawal latency after intraplantar injection of PK2 (2.5 pmole) or saline were expressed as percent change from pretreatment values. PK2 significantly decreased the withdraw latency radiant heat. Two asterisks *P *< 0.01 versus saline treatment.

It has been reported that intraplantar injection of frog Bv8 sensitized the nociceptive response to thermal stimuli in rats [11]. We examined effect of recombinant human PK2 on nociception to thermal stimuli. When 2.5 pmole PK2 was injected into hindpaw, the withdrawal latency of the injected hindpaw to radiant heat decreased to 40.2 ± 5.2% of the basal value and 37.9 ± 10.3% of the contralateral hindpaw. No change of the withdrawal latency was observed when vehicle was injected (Fig. 1). This study reveals that intraplantar injection of PK2 caused a strong and localized hyperalgesia.

### Mobilization of calcium in DRG neurons by PK2

**Figure 2:**
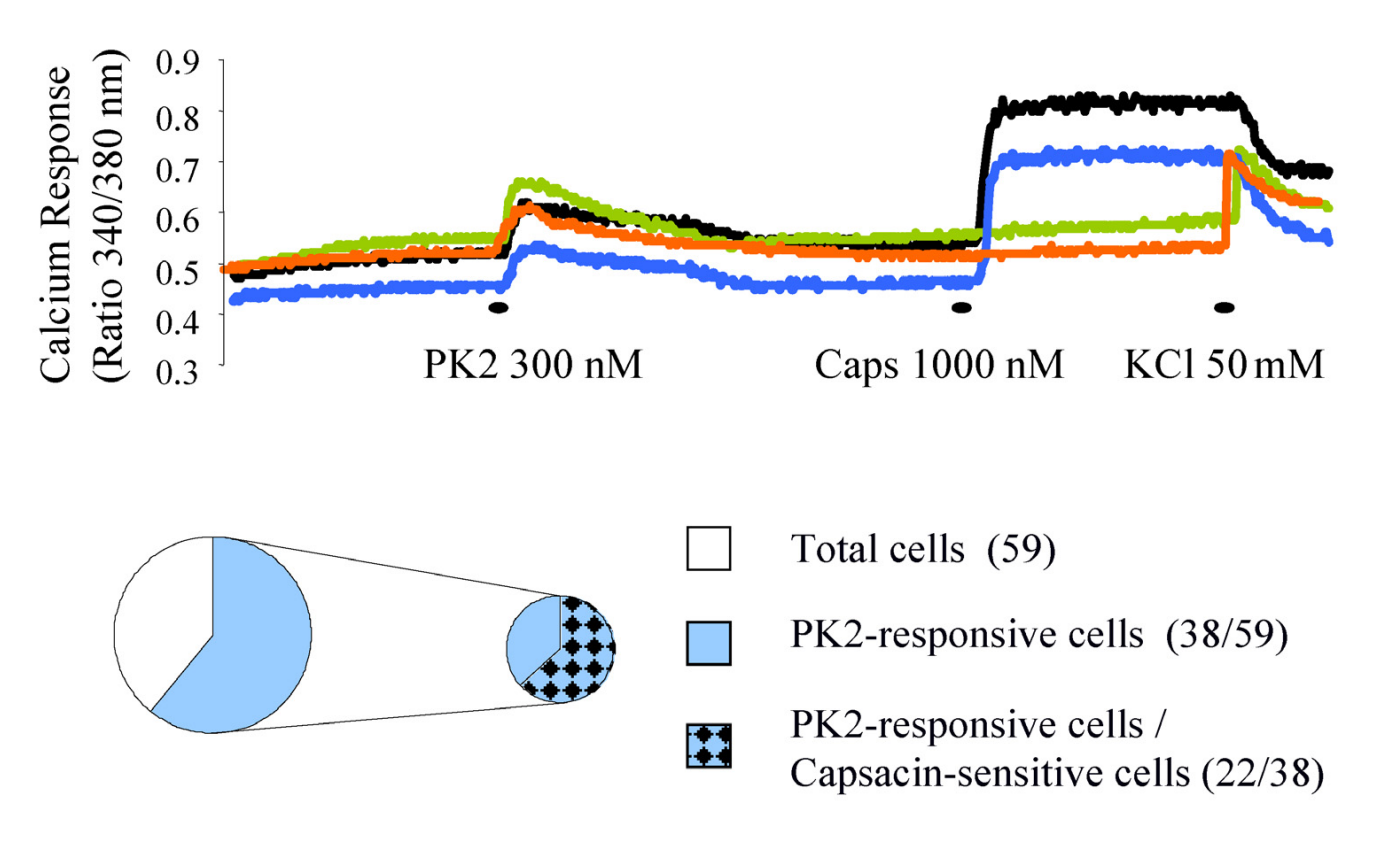
Mobilization of intracellular calcium in dissociated dorsal root ganglion neurons by PK2. The upper panel illustrates examples of responses of individual neurons to recombinant human PK2 (300 nM), capsaicin (1000 nM) and KCl (50 mM). The lower panel shows the distribution of PK2 and/or capsaicin-responsive cells in examined DRG neurons

PK2 is known to mobilize intracellular calcium in cells that express PKRs exogenously [3,4]. We therefore examined the effects of PK2 on [Ca^2+^]_i _in rat DRG neurons. When 300 nM PK2 was applied, [Ca^2+^]_i _increased in about 65% of acutely dissociated DRG neurons (38/59). Furthermore, 22 of the 38 (58%) PK2-responsive DRG neurons exhibited sensitivity to capsaicin (Fig. 2). These results demonstrated the partial overlapping of DRG neurons that respond to PK2 and capsaicin.

### Co-localization of PKRs with TRPV1 in DRG

**Figure 3:**
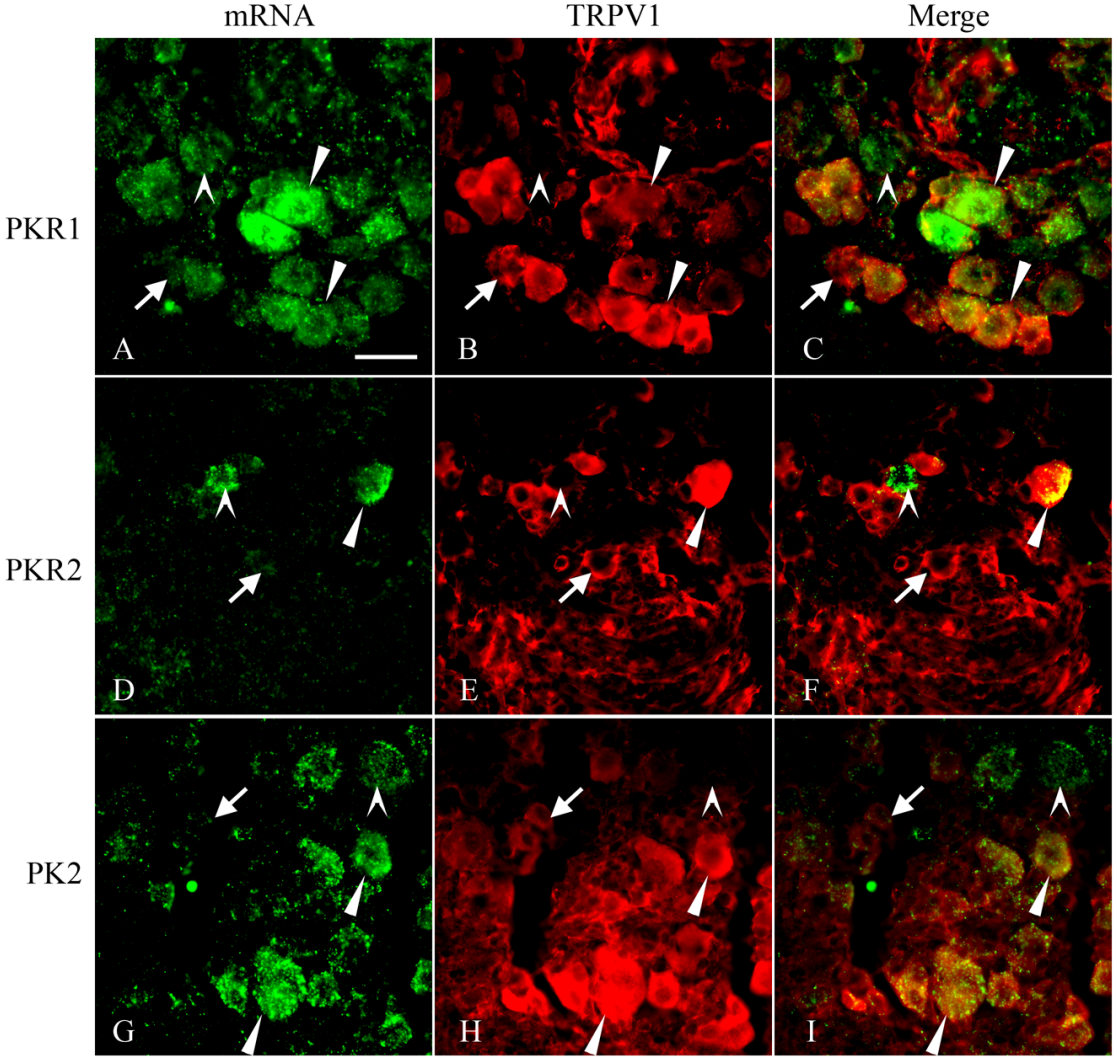
Co-localization of TRPV1 with PKR1, PKR2, and PK2 mRNA in DRG neurons. TRPV1 was revealed by immunostaining (red). PKR1, PKR2 and PK2 expressions were revealed by *in situ *hybridization (green). Arrowhead () showed cells co-express TRPV1 and PKR1, PKR2 or PK2; arrow (→) indicated cells only express TRPV1; and arrowhead () revealed cells only express PKR1 or PKR2 or PK2. Scale bar = 25 μm.

*In situ *hybridization with DIG-labeled riboprobe against PKR1 revealed its expression in many small DRG cells, likely neurons that are involved in nociception. Many of the PKR1-expressing cells also expressed TRPV1 (Fig. 3A–C), although cells that expressed either PKR1 and TRPV1 were also evident. In contrast, only a small number of DRG cells expressed PKR2 (Fig. 3D). Some of the PKR2-postive cells also co-localized with TRPV1, whereas others were not (Fig. 3E–F). These results were consistent with results of calcium mobilization experiments. Interestingly, we also observed the expression of PK2 mRNA in some small DRG cells, some of which were TRPV1-positive (Fig. 3G–I). This result suggested that PK2 might be released from terminals of DRG neurons.

### Attenuated thermal nociception in *PK2-/- *mice

**Figure 4:**
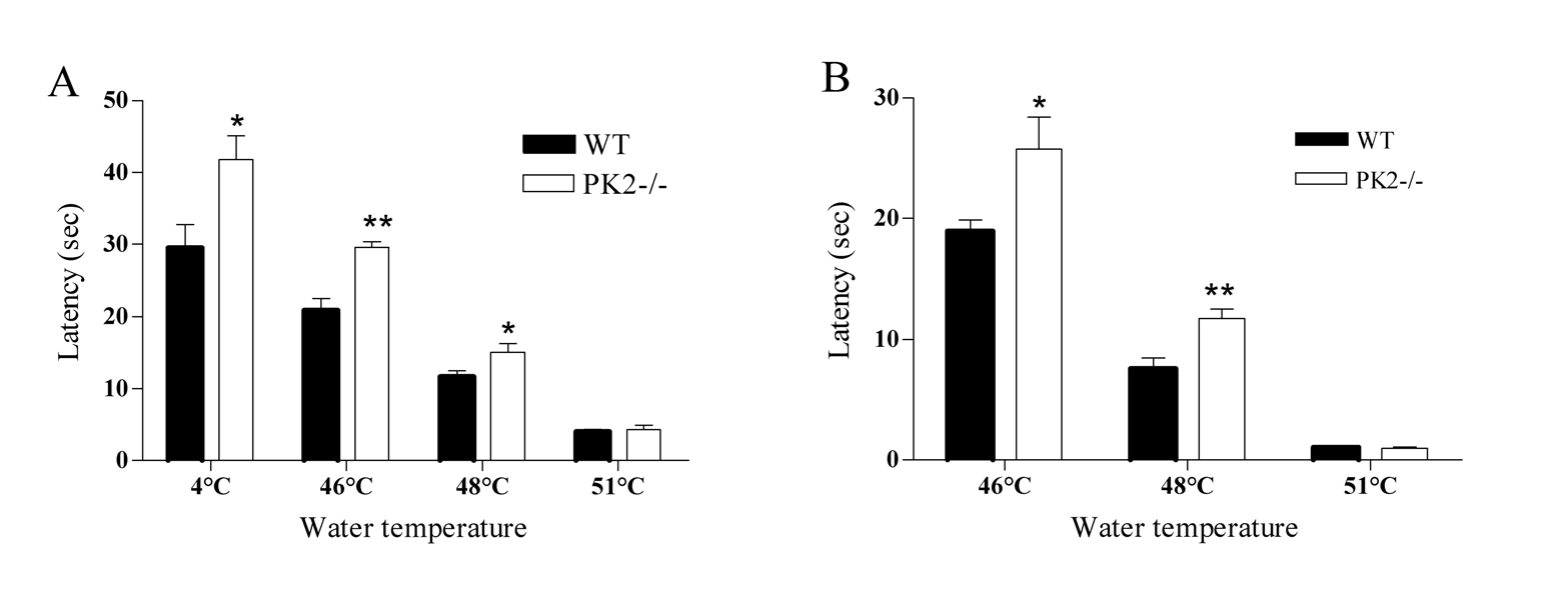
Nociceptive responses of WT and *PK2-/- *mice to thermal stimuli. ***A***, Tail-withdrawal latency from hot and cold water in WT (black bars, n = 9) and *PK2-/- *mice (white bars, n = 8). *PK2-/- *mice exhibited significantly increased tail withdrawal latencies than WT controls at 46°C, 48°C and 4°C. ***B***, Paw-withdrawal latency from hot water. *PK2-/- *mice exhibited significantly increased paw withdrawal latencies than WT controls at 46°C and 48°C. Asterisk, *P *< 0.05; two asterisks *P *< 0.01.

To assess the sensitivity of *PK2-/- *mice to noxious thermal stimuli, we performed the tail and paw immersion tests. The withdrawal latency of the *PK2-/- *mice was comparable to that of wild type (WT) controls when the tail or hindpaw was immersed into 51°C hot water. However, *PK2-/- *mice exhibited significantly increased withdrawal latencies than WT controls at 46°C and 48°C (Fig. 4A–B). *PK2-/- *mice also showed longer withdrawal latencies in cold water (4°C) tail withdrawal test (Fig. 4A). These data indicated that the *PK2-/- *mice had attenuated responses to both hot and cold thermal nociceptive stimuli.

### Impaired nociceptive responses to noxious chemical stimuli in *PK2-/- *mice

**Figure 5:**
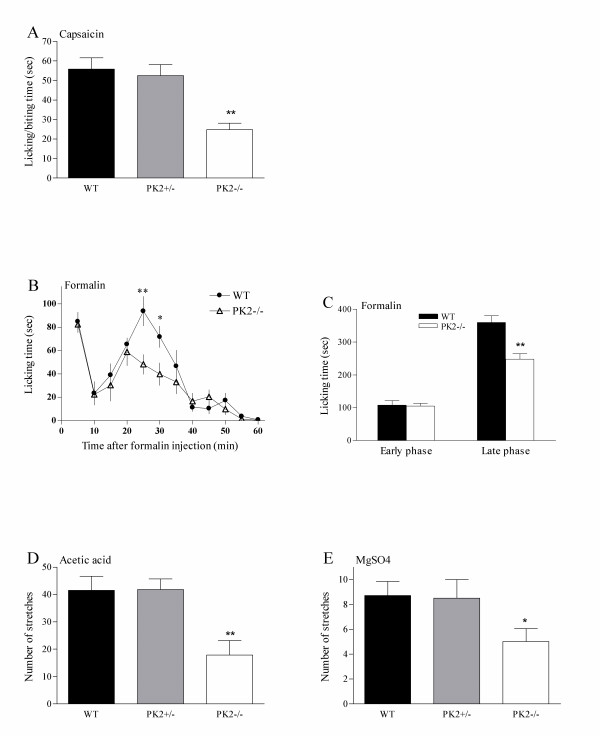
Nociceptive responses of WT and *PK2-/- *mice to noxious chemical stimuli. ***A***. Nociceptive responses to capsaicin. Duration of licking in response to intraplantar injection of capsaicin (3 μg/10 μL) in WT (black bars, n = 8), *PK2+/- *(grey bars, n = 7) and *PK2-/- *mice (white bars, n = 8). ***B***. Nociceptive responses to formalin. Time course of pain behavior induced by intraplantar injection of 5% formalin (20 μL) in WT (filled circles, n = 9) and *PK2-/- *mice (open triangles, n = 8). ***C***. Formalin-induced pain behavior during the early and late phases. ***D***. Visceral pain responses to acetic acid. Abdominal stretching produced by intraperitoneal injection of 0.6% acetic acid (5 μl/g body weight) was recorded. ***E***. Visceral pain response to MgSO_4_. Abdominal stretching produced by intraperitoneal injection of 0.1 mM Mg SO_4 _(10 μl/g body weight) was recorded. All data are mean ± SEM. Asterisk, P < 0.05; two asterisks P < 0.01.

Several nociceptive assays were carried out to investigate the behavioral responses to noxious chemical stimuli in *PK2-/- *mice. Capsaicin is an intensely noxious chemical stimulus that, when injected intraplantarly into the hindpaw, directly activates C-fibers. Intraplantar injection of capsaicin elicited a robust shaking, licking and biting of the hindpaw in WT animals. As shown in Fig. 5A, the nociceptive response to capsaicin was significantly reduced in *PK2-/- *mice. In contrast, *PK2+/- *mice exhibited similar response to capsaicin as WT controls. No difference was observed among three groups of animals when the vehicles were used (data no shown).

Subcutaneous injection of 5% formalin into the ventral hindpaw elicited a biphasic behavioral response, which can be divided into a brief early phase (0–10 min) and a prolonged late phase (10–60 min). While no difference in the early-phase responses was observed between *PK2-/- *mice and WT controls, *PK2-/- *mice exhibited significantly reduced late-phase responses to subcutaneous administration of formalin (Fig. 5B–C).

We then examined the nociceptive responses of the animals to visceral pain induced by intraperitoneal administration of acetic acid or MgSO_4 _solution. In both cases, *PK2-/- *mice exhibited significantly attenuated abdominal stretching response than WT controls, while the *PK2+/- *mice did not differ from WT (Fig. 5D–E). Taken together, all these behavioral experiments showed that the nociceptive responses to noxious chemical stimuli were significantly reduced in *PK2-/- *mice.

### Intact inflammatory response to intraplantar injection of capsaicin in *PK2-/- *mice

**Figure 6:**
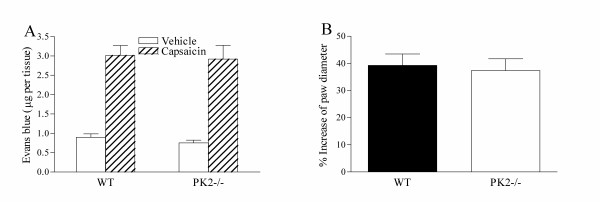
Inflammatory responses of WT and *PK2-/- *mice to intraplantar injection of capsaicin. ***A***. Quantification of Evans blue extravasation 30 min after injection of capsaicin (3 μg/10 μl) or vehicle. ***B***. Percentage of diameter increase of capsaicin injected paws compared to vehicle-injected paws. All data shown are mean ± SEM.

Intraplantar injection of capsaicin induces a marked neurogenic inflammation characterized by increased paw diameter, plasma extravasation and pain [16]. As PK2 mRNA is expressed in inflammatory cells, the capsaicin-induced inflammatory response was examined to determine whether the altered behavioral response to capsaicin in *PK2-/- *mice was related to the difference in inflammatory response. Fig. 6A shows that capsaicin-induced plasma extravasation of Evans blue dye was similar in *PK2-/- *and WT mice. Moreover, comparable increase of paw diameter was also observed in *PK2-/- *and WT mice in response to capsaicin injection (Fig. 6B). These results indicated that the inflammatory response induced by capsaicin was intact in *PK2-/- *mice, even though the pain sensation was attenuated.

## Discussion

In this study, we investigated the role of PK2 in nociception, particularly the inflammatory pain. One striking phenotype of *PK2-/- *mice is the strong reduction in nociception induced by intraplantar injections of capsaicin. This is consistent with the observation that peripheral injection of PK2 and frog Bv8 resulted in potentiation of capsaicin-evoked pain behavior [12]. Intraplantar injection of capsaicin produces nociceptive behaviors in rats and mice with a short-lasting inflammatory response characterized by redness, swelling and plasma extravasation [16]. As PK2 is also expressed in inflammatory cells [9,14], it was intriguing to find out whether the attenuated nociceptive response to capsaicin in *PK2-/- *mice was due to altered inflammation. No difference in inflammatory response to capsaicin, as indicated by Evans blue dye extravasation and equivalent paw edema, was observed between *PK2-/- *and WT mice. Thus, lack of the *PK2 *gene reduced the capsaicin-evoked pain sensation without affecting inflammatory response.

Two receptors for PK2, PKR1 and PKR2, are expressed in some DRG neurons, implying that PK2 may directly activate these PKRs-expressing neurons. Indeed, we observed that PK2 could induce calcium mobilization in isolated DRG neurons. Interestingly, the majority of PK2-responsive DRG neurons were also sensitive to capsaicin, and the percentage of these neurons matches the percentage reduction of capsaicin-induced pain response in the *PK2-/- *mice. Together, these suggest that activation of these PK2-responsive, capsaicin-sensitive neurons may underlie the PK2-induced hypersensitivity. This is supported by our findings that PKRs colocalize with capsaicin-gated ion channel, TRPV1. TRPV1 can be sensitized by phosphorylation through PKC, PKA and Ca^2+^/CaM-dependent kinsase II [17-20]. At sites of tissue injury or inflammation, endogenous factors like bradykinin, prostaglandin and ATP are released and potentiate the TRPV1 mediated nociceptive response through activation of their cognate G protein-coupled receptors [21-24]. Since PKR1 and PKR2, two G-protein coupled receptors, co-express with TRPV1 in the DRG neurons, it is very likely that PK2 would also sensitize TRPV1 by activating PKRs. Patch-clamp experiments revealed that activation of PKRs could potentiate TRPV1 mediated inward current in rat DRG neurons [12]. Our expression results also reveal that PKR1 is the dominant receptor expressed in primary nociceptive DRG neurons, consistent with the genetic study that mice lacking the *PKR1 *gene showed impaired response to capsaicin [13].

Intriguingly, where does the PK2 come from at the site of tissue injury? Recently it has been demonstrated that PK2 is expressed in inflammatory cells and PK2 expression is strongly increased in inflammatory paw skin of mice [9,14]. Particularly, PK2 may be released by neutrophils, macrophages at sites of inflammation [15]. In this study, we showed that PK2 was also expressed in many DRG neurons. Thus, it is probably that PK2 can be released from the terminals of the primary sensory neurons, just like substance P, in neurogenic inflammation.

The TRPV1 channel is known to be a critical molecular transducer of heat and is modulated by protons [19]. In the present study, thermal hyperalgesia was induced by intraplantar injections of PK2. Additionally, mice lacking the *PK2 *gene showed impaired thermal nociception to noxious temperature range from 46 to 48°C, the operating range of C-polymodal nociceptors and TRPV1 [17,25]. These results indicated that PK2 likely acts through a pathway involving TRPV1 function *in vivo*. As there also exist PK2-responsive neurons that are not TRPV1-positive, TRPV1-independent role of PK2 in pain sensation is also likely. Interestingly, *PK2-/- *mice showed attenuated responses to noxious cold. TRPA1, another member of the transient receptor potential (TRP) family of ion channels, is activated by cold (~6°C) and noxious chemicals such as mustard oil [26,27]. Thus, it is possible that PK2 may activate primary sensory neurons via TRPA1. It should also be noted that we have previously shown that PK2 increases the excitability of CNS neurons that express PKR2 via modulating potassium channels [28]. Thus, PK2 may activate primary sensory neurons that express PKR1 and/or PKR2 via different signaling pathways.

Mice lacking the *PK2 *gene exhibited impaired formalin-induced imflammatory pain response. The intact early-phase response in *PK2 *mutant mice suggested that the chemonociceptor terminals mediating the acute phase are intact in the mutant mice.  Central sensitization at the level of spinal cord is thought to be critical for formalin-induced persistent inflammatory pain [29-31]. The reduced late-phase response in *PK2-/- *mice suggested that PK2 might contribute to the underlying changes in central sensitization. Thus, a reasonable explanation for the reduced late-phase response in *PK2-/- *mice could be that, PK2 is released from the central afferent terminals of primary sensory neurons after formalin injection, and, cause central sensitization by activating PKRs in the dorsal horn of spinal cord. This is supported by other findings indicated that intrathecal injection of Bv8 could induce hyperalgesia [11]. Clearly, much remains to be explored for the mechanism of PK2 in pain sensitization.

In conclusion, we have found that PK2 is involved in acute and inflammatory pain. PK2 may modulate sensitization of nociception in the peripheral and central primary sensory afferents during inflammatory pain processing without affecting the inflammation states.

## Materials and methods

### Animals

Homozygous (*PK2-/-*), heterozygous (*PK2+/-) *and wild type littermates (WT) on a C57BL/6J: 129/Ola background were generated as described [32]. All mice were 11 to 20 weeks old and weighed 22–28 g. All procedures regarding the care and use of animals were in accordance with institutional guidelines.

### Behavioral assays

Behavioral studies were performed as described [33]. All animals were acclimated for 60 min in individual plexiglass chambers prior to behavioral experiments.

#### Thermal nociception

For paw-withdrawal to radiant heat tests, mice were placed on the glass surface with 30°C temperature. A mobile radiant heat source located under the glass was focused onto the hindpaw. The paw withdrawal latency was recorded as baseline nociceptive threshold. The effect of the PK2 was calculated as the percentage change relative to baseline threshold. For Tail and paw immersion tests, mice were gently restrained by hand, and the distal half of the tail or one hindpaw was immersed into a water bath. The latency to withdraw the tail or hindpaw was recorded. The test was repeated three times with 1 hr intertrial intervals at each temperature.

#### Chemical nociception

For capsaicin test, capsaicin, 3 μg in 10 μl (Sigma; dissolved in 5% ethanol, 5% Tween-80 and 90% saline), was injected into the dorsal part of the right hindpaw using a 30G needle, after which the animals were placed on a 30°C glass surface and the time spent licking or biting the injecting paw was recorded as the nociceptive score for 10 min after injection. Paw diameter was measured with spring-loaded calipers. For the formalin test, mice received a 10 μl intraplantar injection of 5% formalin and the duration of paw licking or biting was recorded for each 5 min interval during the early phase (0–10 min) and the late phase (10–60 min). For visceral pain, either dilute acetic acid (5 μl/g body weight of 0.6% acetic acid solution) or MgSO_4 _(10 μl/g body weight of 0.1 mM MgSO_4 _solution) was injected into peritoneum, and the number of abdominal stretching were measured for 20 min after acetic acid injection or for 10 min after MgSO_4 _injection.

### Neurogenic plasma extravasation

Neurogenic plasma extravasation was performed as described [13]. Briefly, mice were anaesthetized and injected intravenously with Evans blue (50 mg/kg) into the tail vein. Five minutes later, capsaicin (3 μg/10 μl) was injected into one paw of the animal and vehicle (5% ethanol, 5% Tween 80, and 90% saline) was injected into the other paw. After 30 min the plantar skin of the paw was removed, dried of excess liquid, weighed and incubated in formamide for 24 h at 56°C. Extravasated Evans blue was measured by spectrophotometer at 620 nm.

### Intracellular calcium imaging

Intracellular calcium imaging of DRG neurons was performed as described [34]. Briefly, DRG neurons were acutely dissociated, and loaded with 5 μM fluorescent indicator Fura-2 acetoxymethylester (Fura-2/AM) for 30 min at room temperature. After incubation the neurons were washed several times with normal bath solution (containing in mM: NaCl 125, KCl 1.0, CaCl_2 _5, MgCl_2 _1, glucose 8, and HEPES 20, pH adjusted to 7.4) to remove remaining Fura-2/AM. Coverslips with attached cells were then mounted on a recording chamber and [Ca^2+^]_i _in DRG neurons was monitored by an Attofluor Ratio Vision Digital Fluorescence Microscopy System (Atto Instruments, Rockville, MD). Drugs were delivered by fast perfusion onto neurons using computer-controlled gravity-fed multibarrel perfusion system. Fluorescence was measured with 10 Hz alternating wavelength time scanning with excitation wavelengths of 340 and 380 nm and an emission wavelength of 510 nm. The concentration of Ca^2+ ^was calculated by comparing the ratio of fluorescence at 340 and 380 nm against a standard curve of known [Ca^2+^]_i_.

### *In situ *hybridization and immunohistochemistry

DRGs at the levels of L4 and L5 were removed from wild type mice and fixed in 4% paraformaldehyde and then incubated in 30% sucrose at 4°C overnight. DRGs were then embedded in OCT, and cut at 20 μm on a cryostat. Digoxigenin (DIG)-labeled PK2, PKR1 and PKR2 riboprobes and their sense control riboprobes were made using DIG RNA labeling mix from Roche. *In situ *hybridization using DIG-labeled riboprobes were performed with TSA Plus Fluorescence Kit (Perkin Elmer) as described in the instruction manual. The sections were then incubated with rabbit anti-TRPV1 antibodies (Chemicon International, 1:1000) at 4°C overnight. Cy3-labeled donkey anti-rabbit IgG (Jackson ImmunoResearch, 1:200) was subsequently added. Sections were counterstained with DAPI (Vector Labs) and viewed under a Zeiss fluorescence microscope.
